# Optimisation of a micro-neutralisation assay and its application in antigenic characterisation of influenza viruses

**DOI:** 10.1111/irv.12333

**Published:** 2015-10-13

**Authors:** Yipu Lin, Yan Gu, Stephen A Wharton, Lynne Whittaker, Victoria Gregory, Xiaoyan Li, Simon Metin, Nicholas Cattle, Rodney S Daniels, Alan J Hay, John W McCauley

**Affiliations:** 1 The Francis Crick Institute, Mill Hill LaboratoryLondon, UK

**Keywords:** Antigenicity, haemagglutination inhibition, influenza, micro-neutralisation

## Abstract

**Objectives:**

The identification of antigenic variants and the selection of influenza viruses for vaccine production are based largely on antigenic characterisation of the haemagglutinin (HA) of circulating viruses using the haemagglutination inhibition (HI) assay. However, in addition to evolution related to escape from host immunity, variants emerging as a result of propagation in different cell substrates can complicate the interpretation of HI results. The objective was to develop further a micro-neutralisation (MN) assay to complement the HI assay in antigenic characterisation of influenza viruses to assess the emergence of new antigenic variants and reinforce the selection of vaccine viruses.

**Design and setting:**

A 96-well-plate plaque reduction MN assay based on the measurement of infected cell population using a simple imaging technique.

**Sample:**

Representative influenza A (H1N1) pdm09, A(H3N2) and B viruses isolated between 2004 and 2013

**Main outcome measures and results:**

Improvements to the plaque reduction MN assay included selection of the most suitable cell line according to virus type or subtype, and optimisation of experimental design and data quantitation. Comparisons of the results of MN and HI assays showed the importance of complementary data in determining the true antigenic relationships among recent human influenza A(H1N1)pdm09, A(H3N2) and type B viruses.

**Conclusions:**

Our study demonstrates that the improved MN assay has certain advantages over the HI assay: it is not significantly influenced by the cell-selected amino acid substitutions in the neuraminidase (NA) of A(H3N2) viruses, and it is particularly useful for antigenic characterisation of viruses which either grow to low HA titre and/or undergo an abortive infection resulting in an inability to form plaques in cultured cells.

## Introduction

Influenza viruses evolve constantly to escape human immunity and cause annual epidemics and occasional pandemics. To minimise the impact of influenza, vaccination is the best option, but its effectiveness depends on the degree of antigenic similarity between vaccine viruses and circulating viruses. For over 60 years, the identification of antigenic variants has been determined largely by the haemagglutination inhibition (HI) assay to measure the ability of antibodies, raised against vaccine and reference viruses,^1^ to prevent the attachment of virus to red blood cells (RBCs), a process analogous to the binding of virus to host cell receptors. However, during the past decade, interpretation of HI results has become complicated: either because of changes in receptor binding properties as a result of virus evolution, or due to selection of variants during the isolation and passage of viruses in cell lines or eggs. For example, the loss of the ability of A(H3N2) viruses to agglutinate chicken, and subsequently turkey, RBCs was caused by amino acid substitutions E190D and D225N in the haemagglutinin (HA), occurring around 1990 and 2005, respectively.^2^–^4^ The extremely low avidity of virus for receptors has contributed to the selection of mutations in the neuraminidase (NA) gene during propagation of such viruses in Madin–Darby canine kidney (MDCK) cells.^4^,^5^ Amino acid substitutions flanking the catalytic site of NA (D151G/N or T148I) allow NA to contribute to the binding and agglutination of RBCs in a way that is resistant to inhibition by anti-HA antibodies in post-infection ferret antisera, as assessed by HI assay,^5^ but sensitive to the neuraminidase inhibitor (NAI), oseltamivir carboxylate. It has long been established that during adaptation of influenza B viruses to growth in hens’ eggs, the loss of a glycosylation site in the HA has resulted in different patterns of HI reactivity between egg- and MDCK cell-propagated viruses^6^–^8^ and egg-adaptive changes in influenza A viruses also affect the behaviour of viruses in the HI assay. Furthermore, variation in RBCs derived from different species and individual animals can also affect HI results. Hence, virus neutralisation assays have been used in conjunction with HI to clarify the true antigenic relationships between viruses and to support assessment of the antigenic characteristics of A(H3N2) viruses at the WHO influenza vaccine consultation meetings since 2009.^1^

Although the micro-neutralisation (MN) assay can potentially overcome non-antigenic effects of variation between viruses and between RBCs, most MN assays based on cytopathic effect or ELISA require 100 50% tissue culture infectious dose (TCID_50_) of input virus per well,^9^ presenting a difficulty for the majority of recent A(H3N2) viruses which replicate to low titre in cell culture. Also, quantitation of plaque numbers in a plaque reduction assay is difficult when there is a significant variation in plaque size induced by individual viruses and it is impossible with some currently circulating A(H3N2) viruses that undergo abortive infection and do not form visible plaques.

Here, we describe the application of an improved version of the plaque reduction MN assay to the antigenic characterisation of currently circulating influenza viruses. It is based on measurement of the infected cell population (ICP), in a 96-well-plate format, using a simple imaging method.^10^

## Materials and methods

### Viruses and cells

Influenza viruses were obtained from the Francis Crick Worldwide Influenza Centre. A(H3N2) viruses were propagated in MDCK-SIAT1 cells, and its parent cells were used for propagating A(H1N1)pdm09 and influenza B viruses (both cell lines kindly provided by Dr. M. Matrosovich, University of Marburg, Germany^11^). Mini-pig kidney (MPK) and MDCK-ECACC were obtained from The Pirbright Institute and the European Collection of Cell Cultures, respectively. Viruses were propagated in cell lines incubated at 34°C in Dulbecco's modified Eagle's medium (DMEM), containing 2 μg/ml trypsin (TPCK-treated). Some reference viruses were propagated in 10-day-old embryonated hens’ eggs at 34°C.

### HI assays

Haemagglutination and HI assays were performed according to standard methods using suspensions of guinea pig RBCs (1·0% v/v) for A(H3N2) viruses and turkey RBCs (0·75% v/v) for A(H1N1)pdm09 and type B viruses with post-infection ferret antisera pre-treated with receptor-destroying enzyme (RDE) from *Vibrio cholera*.^12^ Four HA units were used in HI assays of A(H3N2) and type B viruses and 8 HA units in assays of A(H1N1)pdm09 viruses. For A(H3N2) viruses, haemagglutination and HI assays were conducted in the presence of 20 nm oseltamivir carboxylate.^5^

### MN assays

Micro-neutralisation assays were conducted as described previously.^10^ Briefly, doubling dilutions of RDE-treated post-infection ferret antisera were added to confluent cell monolayers in 96-well plates (except for virus and cell control wells) in DMEM; viruses to be tested were then added and plates incubated at 37°C or 34°C for three hours for influenza A and B viruses, respectively. The inoculum was removed and 200 μl/well of overlay medium, containing 1·2% (w/v) Avicel (FMC BioPolymer) and 2 μg/ml trypsin, was added before incubating plates at 37°C for 22 hours (influenza A) or 34°C for 28 hours (influenza B). Cells were fixed with 4% (w/v) paraformaldehyde, stained using a pool of anti-influenza nucleoprotein (NP) mouse monoclonal antibodies, a peroxidase-conjugated goat anti-mouse antibody and TrueBlue™ substrate.^10^ The dried plates were scanned using a flatbed scanner. The ICP in each well, the percentage of positive (infected) cells, was quantified as the percentage of positive pixels using in-house image-processing software,^10^ and the average ICPs were normalised to the virus control for each plate using Excel (Microsoft®). The background measurement for uninfected cell controls was subtracted during data processing. The neutralisation titre was determined as the reciprocal of the antiserum dilution corresponding to 80% reduction in ICP. Linear interpolation was used to estimate titres falling between two adjacent serum dilutions.

The virus (control) inoculum was titrated by serial 5- or 10-fold dilutions. ICPs of triplicate wells were averaged and normalised to the ICP of the wells in which all cells were infected. A virus dilution that caused 20–85% of saturating ICP was chosen, closest to the mid-point. If the ICP did not reach saturation, the maximum titre of virus was used.

### Nucleotide sequence analysis

Following RT/PCR, nucleotide sequences of HA and NA genes were determined using ABI prism BigDye terminator cycle sequencing kits and an ABI 3730XL DNA analyser. Primer sequences are available on request.

## Results

### Optimisation of assay conditions

As the receptor binding properties of different types and subtypes of influenza virus vary, it was important to determine the most suitable cell line for assaying each. MDCK-Parent, MDCK-ECACC, MDCK-SIAT1 and MPK cells were examined for their ability to support virus replication and plaque formation of panels of A(H1N1)pdm09 (2009 -2012), A(H3N2) (1999–2007) and type B (2007–2012) influenza viruses (Table1).

**Table 1:** Comparison of growth and plaque formation in different cell lines

Virus type/subtype*	MDCK-ECACC		MDCK-parent		MDCK-SIAT1		MPK	
	Growth**	Plaque formation**	Growth	Plaque formation	Growth	Plaque formation	Growth	Plaque formation
A(H3N2)	**+**	**+/-**	**+**	**+/-**	**+++**	**+++**	ND	ND
A(H1N1)pdm09	**+++**	**++**	**+++**	**+++**	**++**	**+/−**	**+++**	**+++**
B	**++**	**+**	**+++**	**+++**	**+++**	**++**	**++**	**+/−**

* Comparative experiments, using the same inocula, were performed with several cell-propagated viruses for each type and subtype/lineage (data for B/Victoria- and B/Yamagata-lineage viruses are combined) on different cell lines.

** Growth was assessed by haemagglutination titre, and plaque formation was scored by counting plaques. Plaque formation: +/− = poor: single cells staining positive; + = low: a few small-sized plaques (two to three cells staining); ++ = medium: medium-sized plaques (four to five cells staining); +++ = high: majority of big plaques (> five cells staining).

ND, not done.

A(H3N2) viruses showed the best propagation and plaque formation, as well as a higher frequency of isolation,^13^ on MDCK-SIAT cells, which express increased levels of α 2,6-linked sialic acid on their surface,^11^ and were used in subsequent experiments.

There were no significant differences in haemagglutination titres of A(H1N1)pdm09 when passaged in the different cell lines. However, MDCK-parent cells were used as they yielded higher frequency of isolation of A(H1N1)pdm09 viruses from clinical specimens collected during 2012 and 2013 (data not shown), and larger plaques compared with MDCK-SIAT1 cells.

Titration of influenza B viruses, of both the Yamagata and the Victoria lineages, showed that MDCK-parent cells yielded the highest titres and clearest plaques.

As bovine serum albumin (BSA: Life Technologies BSA Fraction V) might affect the interaction between virus and receptor, particularly in the case of recent A(H3N2) viruses with a low avidity for cell receptors,^4^ its effects were assessed. Inclusion of BSA in the diluent and overlay inhibited the formation of plaques, as shown for A(H3N2) viruses in Figure S1A, and similar effects were observed with A(H1N1)pdm09 and type B viruses (S. Wharton, unpublished observations); therefore, BSA was omitted from the assay. In addition, removal of the inoculum (plus antiserum) before adding the overlay was necessary as the continued presence of serum proteins throughout the plaque assay, as shown for an antiserum raised against an unrelated influenza virus, impaired plaque formation by A(H3N2) viruses (Figure S1B). Similar effects were seen with A(H1N1)pdm09 and B viruses (S. Wharton, unpublished observations).

Incubation of type A viruses at either 37°C or 34°C resulted in no noticeable difference in plaque formation; however, incubation at 34°C resulted in better plaque formation by influenza B viruses. The most appropriate incubation time for both A subtypes was approximately 22 hours post-infection at 37°C, while 28 hours was optimal for type B viruses at 34°C.

### Influence of the amount of input virus on MN titre

ICP was chosen as the criterion for titration of infectivity and neutralisation by antibody,^10^ as it has several advantages over plaque-forming units (PFU): it can accommodate variation in plaque morphology [a single virus stock commonly yields mixtures of large and small plaques, with many small plaques being beyond visual resolution (Figure S2)]; it can be applied to viruses, such as many recent A(H3N2) viruses, showing only single cell abortive infection (the average cell size is 0·65 pixels^10^); and by counting all infected cells, it reduces the influence of plaque merging.

The amount of input virus was standardised so that MN titres calculated for different viruses, from different experiments, or laboratories, could be compared. As many factors can influence the ICP, the range of ICP that gave reproducible results was assessed. Eleven A(H3N2) viruses were examined against five post-infection ferret antisera using up to a 100-fold dilution range of the individual virus stocks in the neutralisation experiments. Results for four representative viruses show that while MN titres can vary markedly depending on the level of ICP (Table2), if the ICP was within the range 20–85%, the variation was on average within a factor of 1·32 ± 0·56, with a 95·4% confidence (2 standard deviations, 45 valid titres at 80% ICP reduction), that is <2-fold variation. For example, the reference antisera gave comparable MN titres for A/Denmark/67/2012 with an input ICP of 27·7% or 56·5%, but substantially reduced titres when the ICP approached saturation at 98%. Similar results were obtained for the other three viruses. A low ICP, due to insufficient input virus for A/Stockholm/30/2012 and A/Stockholm/31/2012, resulted in a substantial increase in MN titre which may be caused in part by a low signal-to-noise ratio. Overall, the results indicated that the 20–85% ICP range represents a good compromise between titre accuracy and the simplicity of the neutralisation assay. This criterion was also supported by data obtained with A(H1N1)pdm09 and type B viruses (Tables S2 and S3).

**Table 2:** Effects of variation in input A(H3N2) virus titre (ICP) on MN titres

Virus	Virus dilution	Virus control**	MN titre (80% reduction in ICP)				
			Post-infection ferret antisera				
			A/Stockholm/18/2012	A/Athens/112/2012	A/Victoria/361/2011E	A/Victoria/361/2011C	A/Texas/50/2012
A/Denmark/67/2012	5 × 10^−5^	27·7%	144	304	224	499	536
	1 × 10^−4^	56·5%	146	373	145	564	718
	**5** × **10**^−4^*	**97·9%**	**65**	**164**	**71**	**204**	**271**
	**5** × **10**^**−3**^	**97·6%**	**12**	**47**	**12**	**63**	**88**
A/Stockholm/39/2012	1 × 10^−4^	28·6%	365	402	229	537	890
	5 × 10^−4^	82·7%	198	312	183	347	683
	**1** × **10**^**−3**^	**96·0%**	**151**	**258**	**130**	**247**	**448**
	**1** × **10**^**−2**^	**97·8%**	**57**	**105**	**50**	**100**	**165**
A/Stockholm/31/2012	**5** × **10**^**−5**^	**18·4%**	**431**	**1041**	**656**	**1951**	**2529**
	1 × 10^−4^	33·6%	267	1397	689	1417	1718
	5 × 10^−4^	82·4%	254	907	265	1191	1666
	**5** × **10**^**−3**^	**99·3%**	**96**	**274**	**96**	**378**	**503**
A/Stockholm/30/2012	**1** × **10**^**−3**^	**12·7%**	**111**	**405**	**173**	**634**	**550**
	5 × 10^−3^	50·2%	90	265	119	377	473
	1 × 10^−2^	77·5%	70	222	92	324	385
	**1** × **10**^**−1**^	**86·0%**	**9**	**46**	**11**	**64**	**98**

* Bold value indicates that the virus control is outside the recommended range (20-85%).

** % of maximum ICP.

For most viruses, there was little difference between relative titres at 50% and 80% reductions in ICP, but MN titres based on 80% reduction were closer to corresponding HI titres and, for comparison studies, were chosen.

### Comparison of MN with HI in antigenic characterisation

#### Influenza A(H1N1)pdm09

Comparable results were obtained for MN and HI assays, as illustrated in Table3 for five viruses, chosen as being representative of A(H1N1)pdm09 viruses circulating in 2012, using three reference antisera. The MN results confirmed the conclusion from the HI data that four of the five viruses were antigenically similar to the A/California/7/2009 vaccine virus; in both assays, all test viruses were recognised at titres within 4-fold of the respective homologous titres by antisera raised against A/California/7/2009 or the A/California/7/2009-like virus, A/Lviv/N6/2009. These two viruses showed similar reactivity in both HI and MN tests, indicating that the HA1 D222G substitution in A/Lviv/N6/2009 does not affect antigenicity. In contrast, A/Hong Kong/6747/2012 was antigenically similar to A/Bayern/69/2009, both viruses carrying a HA1 G155E substitution.

**Table 3:** Comparison of MN and HI titres of A(H1N1)pdm09 viruses

Virus	HA1 subsitutions at positions 155 and 222	Virus passage history**	Post-infection ferret antisera*; MN/HI titres					
			A/California/7/2009		A/Bayern/69/2009		A/Lviv/N6/2009	
			F05/10***		F11/11		FC4/34/09	
			MN	HI	MN	HI	MN	HI
Reference virus								
A/California/7/2009		E6	**1707**	**1280**	1416	640	2044	1280
A/Bayern/69/2009	G155E	M4/S1	394	80	**898**	**160-320**	301	80
A/Lviv/N6/2009	D222G	M4/S1	2074	640	5120	1280	**2260**	**640**
Test virus								
A/Tucuman/11956/2012		M3	3540	2560	2098	640	2461	1280
A/Misiones/6592/2012		M3	3189	1280	2386	640	2848	1280
A/Tucuman/59232/2012		M3	2056	1280	2202	640	2397	1280
A/Corrientes/66335/2012		S2/M1	2262	2560	1359	1280	2146	2560
A/Hong Kong/6747/2012	G155E	M3	536	320	881	160	464	160

* Raised against the viruses indicated; the antiserum number is given; homologous titres for reference viruses are shown in boldface type.

** Passage in eggs (E), MDCK cells (M), or MDCK-SIAT1 cells (S); the number indicates number of passages.

*** Ferret number.

MN titres were generally higher than HI titres. That the homologous titres for the A/Bayern/69/2009 antiserum were lower than the heterologous titres with the A/California/7/2009-like viruses in both HI and MN tests emphasises the comparability of the results between the two assays.

#### Influenza A(H3N2)

In the light of the polymorphisms at positions 148 and/or 151 that enable the NA of A(H3N2) viruses to bind RBCs and influence haemagglutination,^5^ comparisons were made between the MN assay and HI assays conducted with and without added 20 nm oseltamivir carboxylate (Table4).

**Table 4:** Comparison of MN and HI titres of H3N2 viruses

Viruses	NA substitutions at positions 148 and 151**	Virus passage history***	Post-infection ferret antisera*; MN/HI titres											
			A/Stockholm/18/2011			A/Victoria361/2011E			A/Victoria/361/2011C			A/Athens/112/2012		
			MN	HI-Osl†	HI+Osl†	MN	HI-Osl	HI+Osl	MN	HI-Osl	HI+Osl	MN	HI-Osl	HI+Osl
Reference virus														
A/Stockholm/18/2011	D151	C2/S1	**196**	**160**	**160**	58	160	160	74	320	320	174	320	320
A/Victoria/361/2011E	D151	E5	58	160	160	**1739**	**1280**	**2560**	366	640	640	97	320	160
A/Victoria/361/2011C	D151X	M2/S3	133	80	320	248	80	320	**604**	**160**	**1280**	413	320	640
A/Athens/112/2012	D151	M2	198	160	320	84	160	320	182	640	640	**287**	**640**	**640**
Test virus														
A/Texas/50/2012	D151	E7	361	1280	1280	548	1280	1280	817	2560	2560	749	2560	2560
A/Berlin/165/2012	D151	C2/S1	317	320	320	202	160	160	607	1280	1280	561	640	640
A/Roma/02/2013	D151	C1/S1	171	160	160	201	160	160	701	320	320	444	640	640
A/Stockholm/31/2012	D151	C2/Mx/S1	267	160	320	689	160	320	1417	640	640	1397	640	640
A/Cairo/138/2012	D151	C1/S1	204	320	160	276	320	320	718	1280	1280	655	1280	640
A/Stockholm/40/2012	D151D>G	M1/S1	115	40	160	134	80	160	408	160	640	364	320	640
A/Denmark/67/2012	T148T>I, D151D=N	M3/S1	144	80	320	224	80	160	499	160	1280	364	320	640
A/Denmark/68/2012	T148T<I, D151D>N	Cx/S1	123	80	320	138	80	160	428	320	1280	447	640	1280

* Raised against the viruses indicated; homologous titres for reference viruses are shown in boldface type.

** The T148I substitution results in the loss of a potential N-linked glycosylation site at N146; the D151N substitution results in the gain of a potential N-linked glycosylation site at N151; the relative proportions of amino acids at polymorphic positions are indicated (< less than, > greater than, = equal). X indicates a mixture of amino acids D, G, N, S at position 151.

*** Passage in eggs (E), MDCK cells (M), MDCK-SIAT1 cells (S), or cell type not specified (C); the number indicates the number of passages (x, unknown number).

† Osl indicates 20 nm oseltamivir carboxylate included (+) or absent (−) in the HI assays.

As shown for A/Stockholm/40/2012, A/Denmark/67/2012 and A/Denmark/68/2012, viruses carrying a polymorphism in NA at position 148 or 151 in the catalytic site had lower HI titres (2- to 4-fold) in the absence than in the presence of oseltamivir carboxylate, as observed for viruses in our previous study,^5^ and lower than the corresponding MN titres, with the exception of results obtained with the antiserum raised against A/Athens/112/2012 (Table4).

In the MN assay, antisera raised against cell-propagated reference viruses, A/Stockholm/18/2011, A/Athens/112/2012 and A/Victoria/361/2011C, reacted well (reductions in titres were < 2-fold) with all seven cell-propagated test viruses and the egg-propagated A/Texas/50/2012, indicative of antigenic similarity between test and reference viruses. However, for the antiserum raised against egg-propagated A/Victoria/361/2011E, there were up to 12-fold reductions in MN titre compared to the homologous titre, which is similar to the differences in titres of up to 16-fold in the HI assays carried out in the presence or absence of oseltamivir carboxylate. These differences relate to A/Victoria/361/2011E having acquired two HA1 egg-adaptation substitutions, H156R/Q and G186V.

#### Influenza B viruses

Viruses of the two influenza B lineages were readily discriminated by the MN assay, as in the HI assay, despite some low level cross-reactivity of particular post-infection ferret antisera (Table S1). The antigenic relationships of viruses within each of the lineages were determined in parallel by MN and HI assays, utilising both egg-propagated and cell-propagated viruses, notably to assess and compare the influence of the presence or absence of a glycosylation site at asparagine 197 (Victoria-lineage) or 196 (Yamagata-lineage) which is commonly lost on passage in eggs.^6^,^7^,^14^

Both HI and MN assays clearly distinguished the B/Brisbane/60/2008 vaccine virus and the reference virus B/Malta/63674/2011, from the earlier vaccine virus B/Malaysia/2506/2004 using antisera raised against these egg-propagated viruses (Table5). However, test viruses collected in 2011 or 2012 and propagated exclusively in cell culture, and the cell-propagated reference virus B/Hong Kong/514/2009, were poorly recognised by antisera raised against the contemporary egg-propagated reference viruses in HI assays, with titres 4- to ≥64-fold lower than the titres of the antisera with their homologous viruses, as expected.^8^ Conversely, antiserum raised against cell-propagated B/Hong Kong/514/2009 reacted poorly with the egg-propagated viruses, but gave titres within 2-fold of the homologous HI titre with the test viruses.

**Table 5:** Comparison of MN and HI titres of influenza B viruses (Victoria-lineage)

Viruses	Genetic group-amino acid identities at HA1 positions 197-199	Virus passage history***	Post-infection ferret antisera*; MN/HI titres**							
			B/Malaysia/2506/2004		B/Brisbane/60/2008		B/Hong Kong/514/2009		B/Malta/636714/2011	
			MN	HI	MN	HI	MN	HI	MN	HI
Reference virus										
B/Malaysia/2506/2004	0-NEI	E9	**137**	**160**	66	80	15	<	68	80
B/Bribane/60/2008	1A-SET	E6	10	40	**208**	**640**	17	40	109	320
B/Hong Kong/514/2009	1B-NET	M2/S1	30	<	101	80	**164**	**160**	72	80
B/Malta/636714/2011	1A-SET	E5	32	40	364	640	35	40	**194**	**320**
Test virus										
B/Denmark/15/2012	1A-S/NET†	M3	33	<	228	40	64	80	126	20
B/Sweden/3/2011	1A-NET	C2/M1	29	10	86	40	84	160	42	40
B/Hevecam/17457 GVFI/2011	1A-NET/N†	M2	32	<	281	<	64	80	206	20
B/Yaounde/17518 GVFI/2011	1B-NET	M2	17	<	78	<	81	80	65	20
B/Ukraine/5376/2012	1A-NET	C1/M1	15	<	54	40	50	80	23	10

* Raised against the viruses indicated; homologous titres for reference viruses are shown in boldface type.

** < = less than 10.

*** Passage in eggs (E), MDCK cells (M), MDCK-SIAT1 cells (S) or cells of unspecified type (C); the numbers indicate the number of passages.

† The S/N polymorphism at position 197 and the T/N polymorphism at position 199 result in partial loss of a N-linked glycosylation site at position 197.

The differences observed between cell-propagated and egg-propagated viruses were generally less marked in MN assays with all viruses being recognised by the antisera within 8-fold of the titres for the homologous viruses (Table5). Notably, two test viruses, B/Denmark/15/2012 and B/Hevecam/17457 GVF1/2011, were recognised with MN titres similar to the homologous titres for two egg-propagated reference viruses (B/Brisbane/60/2008 and B/Malta/636714/2011). Both carried an amino acid polymorphism in the 197-199 glycosylation sequon, S/N197 in B/Denmark/15/2012 and N/T199 in B/Hevecam/17457 GVF1/2011, which would result in a partial loss of glycosylation at position 197. In the MN assay, as in the HI assay, antiserum raised against the cell-propagated B/Hong Kong/514/2009 reference virus recognised all test viruses at titres within 4-fold of the titre for the homologous virus.

The HA genes of recently circulating B/Yamagata-lineage viruses fall into two clades.^15^ Like viruses of the B/Victoria-lineage, cell-propagated viruses of the B/Yamagata-lineage were recognised poorly in HI assays by antisera raised against egg-propagated viruses, at 2- to 16-fold lower titres compared to the respective homologous titres, and these antisera did not discriminate between viruses of the two clades (Table6). In contrast, clade 2 viruses were well recognised by the antiserum raised against the cell-propagated clade 2 reference virus B/Estonia/55669/2011, at HI titres within 4-fold of the homologous titre; conversely, clade 3 viruses were recognised at 8- to 64-fold lower HI titres by this antiserum. Discrimination between test viruses in the two clades was less marked in the corresponding MN assays. However, the antiserum raised against egg-propagated B/Wisconsin/1/2010 (clade 3) appeared to discriminate better, giving higher titres with clade 3 viruses than with clade 2 viruses. B/Denmark/8/2012 (clade 3) appeared more closely related to clade 2 than clade 3 viruses; the reason is unknown, but it is noteworthy that the HA gene of B/Denmark/8/2012 did not encode K298E and E312K substitutions, common in clade 3 viruses, but carried amino acids typical of clade 2 viruses at these positions. The antiserum raised against egg-propagated B/Massachusetts/02/2012 did not distinguish between test viruses of clade 2 and 3 in either HI or MN assays.

**Table 6:** Comparison of MN and HI titres of influenza B viruses (Yamagata-lineage)

Viruses	Genetic clade-amino acid identities at HA1 positions 196-198	Virus passage history**	Post-infection ferret antisera*; MN/HI titres							
			B/Florida/4/2006		B/Wisconsin/1/2010		B/Estonia/55669/2011		B/Massachusetts/02/2012	
			MN	HI	MN	HI	MN	HI	MN	HI
Reference virus										
B/Florida/4/2006	1-DKT	E7	**540**	**640**	161	160	61	160	796	640
B/Wisconsin/1/2010	3-DKT	E5	844	160	**330**	**160**	41	10	450	320
B/Estonia/55669/2011	2-NKT	M2	71	160	24	80	**152**	**640**	155	160
B/Massachusetts/02/2012	2-DKT	E7	663	320	133	160	ND***	80	**734**	**640**
Test virus										
B/Denmark/8/2012	3-NKT	M3	170	80	52	10	159	80	231	160
B/Denmark/3/2012	3-NKT	M4	239	40	134	40	73	20	99	80
B/Ireland/13M98449/2013	3-NKT	Cx	187	40	129	40	32	40	112	80
B/Hong Kong/432/2013	3-NKT	M2	203	80	140	40	25	40	130	160
B/Stockholm/8/2012	2-NKT	M2	226	80	136	80	95	640	95	80
B/Paris/2327/2013	2-NKT	M2	213	40	77	40	94	160	106	80
B/Cameroon/13v-2053/2013	2-NKT	M2	120	40	51	20	63	320	100	80
B/Moldova/462/2013	2-NKT	M2	143	40	48	20	149	640	91	80

* Raised against the viruses indicated; homologous titres for reference viruses are shown in boldface type.

** Passage in eggs (E); MDCK cells (M); cells of unspecified type (C); numbers indicate the number of passages (x  =  unknown number).

*** Not done.

Overall, the results of the MN assay appeared to be less affected by the passage history of type B viruses than those of the HI assay.

## Discussion

Comparisons of results of MN and HI assays demonstrated the usefulness of the improved MN assay in complementing the HI assay when determining antigenic relationships among recently circulating human influenza A and B viruses, and interpreting the impact of non-antigenic variation (for example, changes in receptor binding specificity, affinity or avidity) on HI titre.

In addition to selection of the best cell line to support plaque formation (MDCK-parent cells for A(H1N1)pdm09 and type B viruses, and MDCK-SIAT1 cells for A(H3N2) viruses) imaging-aided quantitation of the ICP, which detects all infected cells from visible plaques to single infected cells, greatly enhanced the consistency and speed of the MN assay for antigenic characterisation. As long as the ICP was between 20% and 85% of the total cell population, the results from different neutralisation experiments were comparable (within 2-fold for replicate assays).

Titration of antibodies by HI and neutralisation are not always comparable,^16^ and the sensitivity and the efficiency of the HI assay can be affected by the species of RBCs used, exacerbated by individual animal variation, and the cell substrate used for virus isolation and propagation.^2^,^5^ The major antigenic sites on the HA are located around the receptor binding site,^17^ and HI measures the inhibition of virus binding to sialic acid receptors present on RBCs. However, other antibodies present in convalescent serum can bind elsewhere on the HA, notably those that bind to the stem of the HA.^18^,^19^ MN assays have the potential to detect the effects of a wider range of antibodies than HI and therefore have the ability to reflect more comprehensively the antigenic similarities or differences between viruses.

Results of MN were generally consistent with and confirmed those of HI, notably in revealing antigenic differences between antigenic drift variants of recent type A and type B vaccine viruses, and antigenic changes caused by culture-selected or sporadic changes, such as the G155E substitution in HA1 of certain A(H1N1)pdm09 viruses. However, differences can occur due to alteration in receptor binding, as exemplified by recent A(H3N2) viruses. Amino acid polymorphisms at positions 148 or 151 of NA, which cause NA-dependent binding to sialic acid receptors, enhance ‘apparent’ HA titres. As anti-HA antibodies do not block such binding, reductions in HI titres might be interpreted erroneously as differences in the antigenicity of the HA.^5^ Thus, MN assay results paralleled those of HI only when oseltamivir carboxylate was included in the HI test, indicating that the MN assay was less affected by NA-dependent binding and reflected antigenic differences more accurately with the cells used here.

Influenza B viruses propagated in eggs commonly select HA1 substitutions which result in the loss of an N-linked glycosylation site.^6^–^8^ Glycans attached to Asn-196/7 (Yamagata/Victoria lineage) in the HA can interfere with binding to receptors^20^ and can also alter antigenicity.^7^,^20^,^21^ Loss of these glycans, on the periphery of the receptor binding site, results in the exposure of a highly immunogenic site^6^ that is masked by the glycan in cell-propagated viruses. Hence, while influenza B viruses that have either lost or become polymorphic in the glycosylation site exhibit high HI titres with antisera raised against egg-propagated viruses, most cell-propagated viruses (of both lineages) were recognised at ≥ 8-fold lower HI titres. The differences in MN titres between egg-propagated and cell-propagated viruses were lower, 2- to 8-fold, showing that effects of glycosylation were less pronounced in the MN assay.

As the majority of influenza vaccines are manufactured using egg-propagated B viruses that have lost glycosylation at Asn-196/7 of HA1, the influence on immunogenicity and/or antigenicity of influenza B viruses is of particular importance.^6^–^8^ It is also evident that cell-propagated reference viruses, and the corresponding post-infection ferret antisera, are more appropriate than egg-based reagents for determining the antigenic relationships among influenza B viruses, whether performed by HI or MN assays.

In conclusion, this study has established experimental parameters for a robust MN assay and reinforces the importance and reliability of MN results in supporting HI data for detailed antigenic analyses of currently circulating influenza viruses, notably for the biannual selection of viruses for inclusion in human influenza vaccines.

## Supporting Information

**Figure S1.** Effects of BSA (A) or non-related antiserum (B) on plaque formation by A/Brisbane/10/2007(H3N2) in MDCK-SIAT1 cells.

**Figure S2.** Variation in plaque size and morphology

**Table S1.** Lack of cross-neutralisation between influenza B viruses of the two lineages.

**Table S2.** Effects of variation in input A(H1N1)pdm09 virus titre (ICP) on MN titres.

**Table S3.** Effects of variation in input type B virus titre (ICP) on MN titres.
