# Supplementary figures and images for: Optimisation of a micro-neutralisation assay and its application in antigenic characterisation of influenza viruses
Source: Influenza Other Respir Viruses. 2015 Oct 13;9(6):331–40. doi: 10.1111/irv.12333 (PMC4605415; doi:10.1111/irv.12333)

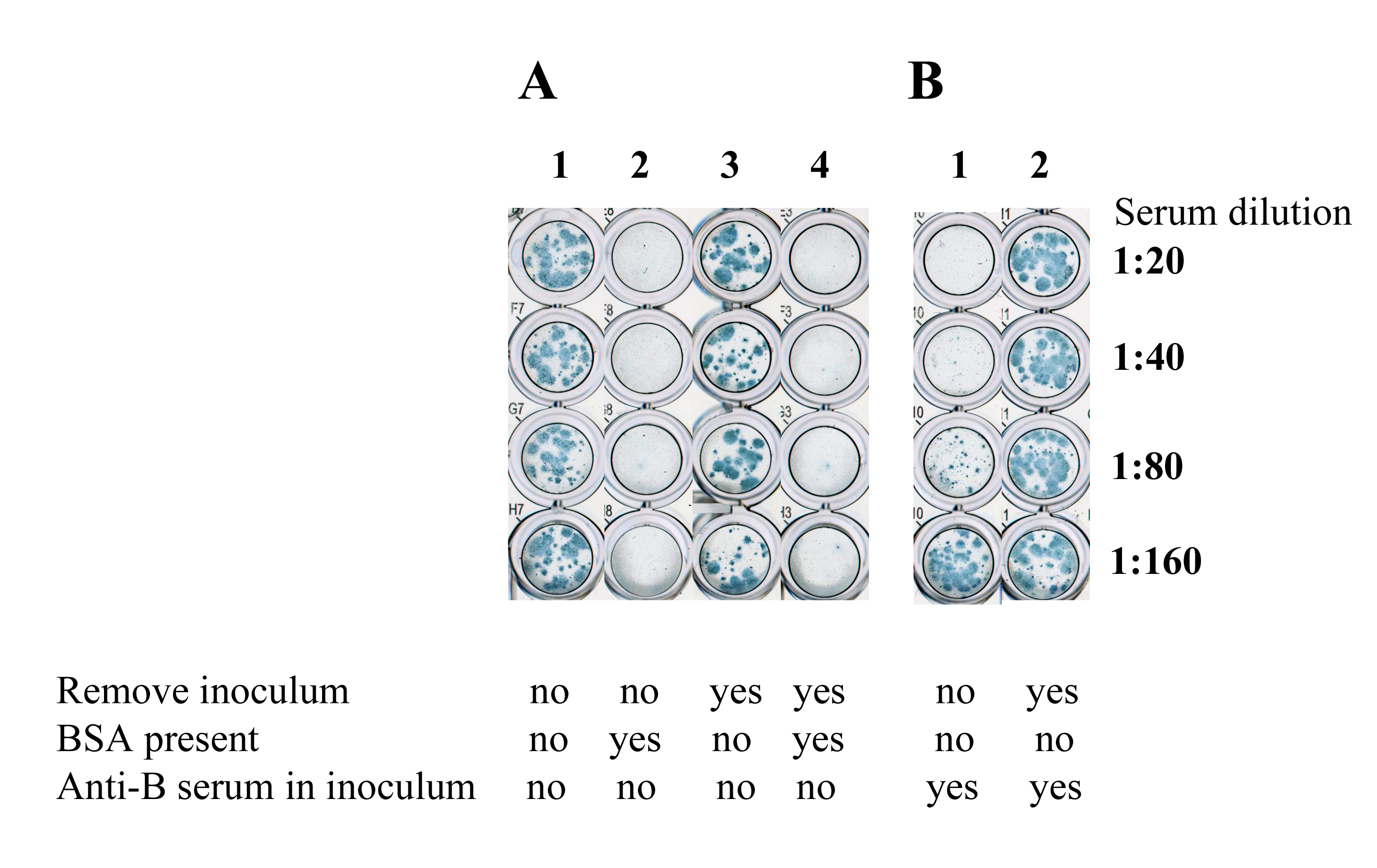

Supplement: Supplementary file 1 — Figure S1. Effects of BSA (A) or non-related antiserum (B) on plaque formation by A/Brisbane/10/2007(H3N2) in MDCK-SIAT1 cells. [file irv0009-0331-sd1.tif]

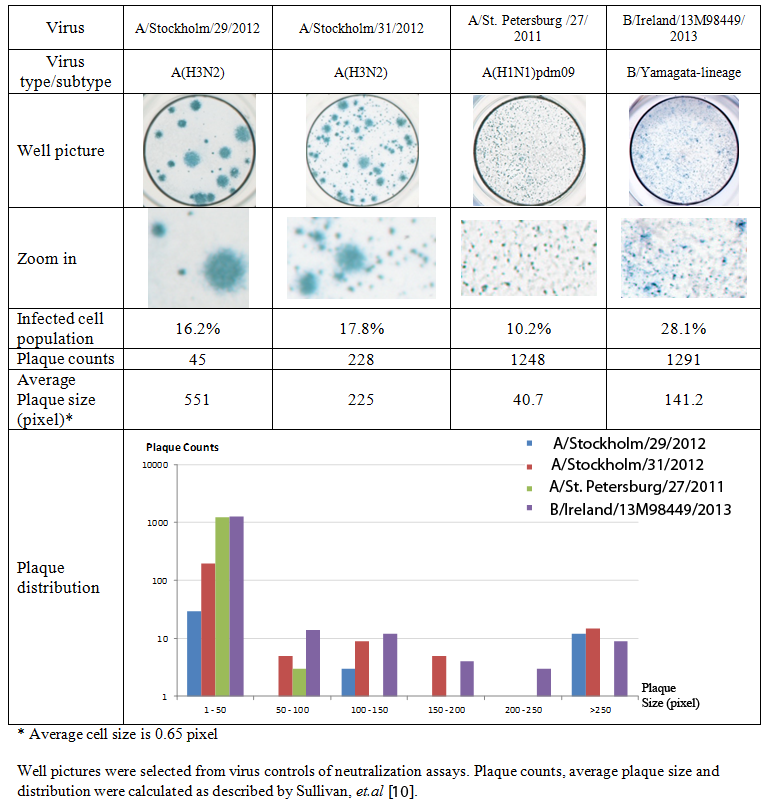

Supplement: Supplementary file 2 — Figure S2. Variation in plaque size and morphology [file irv0009-0331-sd2.tif]
