# Supplementary material for: Optimisation of a micro-neutralisation assay and its application in antigenic characterisation of influenza viruses
Source: Influenza Other Respir Viruses. 2015 Oct 13;9(6):331–40. doi: 10.1111/irv.12333 (PMC4605415; doi:10.1111/irv.12333)
Supplement: Supplementary file 6 [file irv0009-0331-sd6.docx]

Supplementary Information

Table S1. Lack of cross-neutralization between influenza B viruses of the two lineages

Since the early 1980s, two antigenically and genetically distinct lineages of influenza B viruses, B/Victoria and B/Yamagata, have co-circulated in the human population ^22^. To assess cross-reactivity in the MN assay between viruses of the two lineages, five B/Victoria-lineage viruses were tested against post-infection ferret antisera raised against five B/Yamagata-lineage viruses and *vice versa* (Table S1). The five B/Victoria-lineage test viruses gave MN titres of < 20 with all five antisera raised against B/Yamagata-lineage reference viruses, compared to the homologous titres of between 64 and 734 for the five B/Yamagata-lineage viruses. Only in one instance, B/Ireland/87829/2012 with antiserum raised against B/Novosibirsk/1/2012C, was the reduction (3.6-fold) less than ~6-fold or greater. All five B/Yamagata-lineage viruses also gave titres of ≤ 20 with four of five ferret antisera raised against B/Victoria-lineage viruses, compared to homologous titres of between 68 and 208 for the four B/Victoria-lineage viruses. However, two egg-propagated B/Yamagata-lineage viruses, B/Florida/4/2006 and B/Massachusetts/02/2012, showed higher cross-reactivity with antiserum raised against B/Brisbane/60/2008 (of the B/Victoria-lineage) yielding respective titres in the MN assay approximately 7- and 3-fold lower than the homologous titre. In comparative HI tests cross-reactivity of the B/Brisbane/60/2008 antiserum with these two viruses of the B/Yamagata-lineage was 8-fold and 32-fold lower than the titre with the homologous virus.

Table S2. Effects of variation in input A(H1N1)pdm09 virus titre (ICP) on MN titres

Table S3. Effects of variation in input type B virus titre (ICP) on MN titres

Figure S1. Effects of BSA (A) or non-related antiserum (B) on plaque formation by A/Brisbane/10/2007(H3N2) in MDCK-SIAT1 cells

Figure S2. Variation in plaque size and morphology
